# Studying nursing at Australian satellite university campuses: A review of teaching, learning and support

**DOI:** 10.1111/ajr.12741

**Published:** 2021-05-13

**Authors:** Catherine Hays, Susan Devine, Nualnong Wongtongkam, Beverley Glass

**Affiliations:** ^1^ College of Public Health, Medical and Veterinary Sciences James Cook University Townsville QLD Australia; ^2^ Centre for Rural and Remote Health James Cook University Mount Isa QLD Australia; ^3^ College of Medicine and Dentistry James Cook University Douglas QLD Australia

**Keywords:** nursing education, rural and remote education, rural workforce, teaching and learning, undergraduate teaching

## Abstract

Distribution of the Australian health workforce is uneven, with the majority of health professionals favouring metropolitan areas over rural and remote regions. Although nurses account for the largest proportion of the Australian rural and remote health workforce, difficulties with staff recruitment and retention can impact the health care outcomes of these vulnerable populations. Satellite university campuses that offer undergraduate nursing programs might therefore contribute to a more sustainable rural and remote nursing workforce. This narrative literature review aimed at investigating the barriers and enablers that affect students enrolled at satellite nursing campuses, education delivery methods and academic and non‐academic strategies employed to enhance the student learning experience. The literature was reviewed across 6 health and education databases. After screening, 12 articles met the inclusion criteria and were analysed, and the data were synthesised using a thematic approach. Three themes arose from the review: student characteristics and associated barriers and enablers to studying nursing at a satellite campus; teaching strategies and learning experiences; and academic and pastoral support. Students studying at satellite campuses were found to have different education experiences and faced challenges unique to their context; however, home support networks and small class sizes were seen as enabling factors. Education delivery methods and support strategies varied depending on remoteness and resources available. Consideration of the factors that affect satellite campus nursing students has the potential to increase student satisfaction and retention, which could result in a more sustainable rural and remote nursing workforce.

**What is already known on this subject?**

- Nurses and midwives comprise over two‐thirds of the remote nursing workforce; however, difficulties in recruitment and retention in these regions have resulted in instability of the rural and remote nursing workforce, which places nurses and patients at risk and
- Satellite campus nursing graduates are more likely to live and work in rural and remote areas, which might strengthen the rural and remote nursing workforce and improve health outcomes for their local communities

**What this study adds:**

- Students studying nursing at satellite campuses experience barriers and enablers to university participation that are unique to their regional, rural and remote contexts
- Satellite nursing campuses employ a variety of education delivery methods, including face‐to‐face teaching, videoconference and recorded lectures and
- Campuses might offer academic support programs tailored to their student population, which align with local cultural and academic needs.

## 1 INTRODUCTION

Australia's health workforce is distributed unevenly, with the vast majority of health professionals working in metropolitan areas, while rural and remote regions are underserved.^1^ Nurses and midwives form an integral part of the rural and remote health workforce, accounting for 68% of registered health professionals in remote and very remote areas.^2^ However, difficulties in recruitment and retention of nurses in these areas have a direct effect on health care outcomes for rural and remote populations, who face higher rates of mortality, chronic diseases and total disease burden compared with their metropolitan counterparts.^2^, ^3^, ^4^ These health outcomes are influenced by reduced access to specialist health services, increased social disadvantage and higher prevalence of health risk factors including smoking, alcohol consumption and obesity.^4^, ^5^ High turnover rates of nurses and other health professionals, and a reliance on temporary staffing such as agency staff contribute to poorer continuity of care, reduced health outcomes and decreased staff safety.^6^

Undergraduate health students' decisions to work rurally might be positively influenced by rural origin, rural clinical placements and attending university campuses in regional, rural and remote regions of Australia.^1^, ^3^, ^7^, ^8^, ^9^ There is evidence that non‐metropolitan campus graduates and those with rural associations are more likely to work rurally and that prior rural life experience produces ‘rural‐ready' nursing graduates.^8^, ^9^ In 2015, there were approximately 60 satellite university campuses (university campuses located remotely from the main university, also known as regional, rural, remote or branch campuses) across Australia, delivering higher education opportunities to communities throughout the country.^10^ Satellite campuses generally employ non‐traditional teaching methods and have fewer resources and facilities associated with metropolitan universities.^10^, ^11^

These satellite campuses also have the potential to increase university enrolments of disadvantaged populations, including Aboriginal and Torres Strait Islander people, and those from rural and remote areas or low socio‐economic backgrounds.^11^ Students enrolled at satellite campuses are also more likely to be mature‐aged, have families and be the first in their family to attend university.^11^, ^12^ The availability of locally based higher education programs can encourage rural residents, who would not otherwise have access to pursue university education. The social, emotional and financial stress of leaving friends and family and relocating for university can also be avoided.^9^

However, satellite campuses operate with varying degrees of success, from high student satisfaction levels and increased enrolments to high rates of course withdrawals and community disengagement.^10^ Student experience, course delivery methods and support systems in place at satellite university campuses might differ from the more ‘traditional’ systems in place at a conventional university campus.^11^ Consideration of the local community, industry and existing challenges can result in a more appropriate and positive educational experience for students.^11^ The support and flexibility of satellite university campuses improves the sustainability of their local community by contributing to the skilled workforce.^11^

Satellite campuses play an important part in providing equitable access to higher education and, in the context of nursing and other health disciplines, can lead to a more sustainable health workforce and positive health outcomes for rural and remote communities.^8^ However, the effective delivery of education via appropriate methods, support from academic and non‐academic staff, and an understanding of the local community can have a high level effect on the student experience.^8^

It is important to investigate and consider current models of teaching, learning and support, and the successful strategies used to overcome the barriers faced by rural and remote undergraduate nursing students. An increased understanding of these factors has the potential to influence future course delivery and government strategies to create a more sustainable rural and remote primary health workforce. This narrative literature review thus aimed at investigating methods of education in addition to student experiences of teaching, learning and support at satellite campuses, with a focus on undergraduate nursing education by exploring the following: (a) the barriers and enablers of studying at a satellite campus; (b) how teaching and learning strategies used at satellite campuses differ from more traditional modes of learning; and (c) how support is provided to students at a satellite campus to enhance their learning experience.

## 2 METHODS

This narrative review was undertaken according to the methodologies of Ferrari^13^ and Green et al^14^ Due to the nature of the topic, a systematic search of both health and education databases was undertaken, including the Cumulated Index to Nursing and Allied Health Literature, MEDLINE (Ovid), Informit (Health and Education streams), Scopus, EmCare and Education Resources Information Centre. Keywords included terms such as ‘satellite university campus, undergraduate students, education, nursing’, and related terms, listed in Table 1. Inclusion and exclusion criteria for the review are listed in Table 2.

**TABLE 1:** Keyword search terms

“satellite site” OR “satellite campus” OR “remote university campus” OR “remote campus” OR “remote university site” OR “remote site” OR “regional university” OR “regional site” OR “regional campus” OR “rural site” OR “rural campus” OR “Off‐campus” OR “regional college” OR “rural college” OR “small college” OR “multicampus college” OR “rural education”	AND
“undergraduate students” OR “university” OR “tertiary education” OR “higher education”	AND
Teaching OR learning OR strategies OR experiences OR opinions OR perceptions	AND
Nurse OR nursing OR “nursing school”	

**TABLE 2:** Inclusion and exclusion criteria

Inclusion criteria	Exclusion criteria
All publication dates Peer‐reviewed Full‐text available Original research English language only Undergraduate nursing education Regional, rural or remote locations Satellite university campus (or related terms)	Not peer‐reviewed Non‐Australian studies Postgraduate nursing education External or off‐campus study Metropolitan locations Study design (thesis, dissertation, literature review)

Eight hundred and ninety‐nine papers resulted from the search. After removal of duplicates, 491 remained. All titles and abstracts were reviewed for relevance to the scope of the review (Table 2). Initially, no restrictions were applied based on study design or geographical location. Papers were included if they contained at least 2 of the following criteria: nursing education; regional, rural or remote locations; and satellite campus (or related terms). Following abstract review, the remaining 24 papers were assessed for relevance to the current review as outlined in Ferrari^13^ and Green et al.^14^ Seventeen papers were excluded based on project type (thesis/dissertation, literature review) and relevance to the review (postgraduate study, off‐campus/distance education). Three non‐Australian studies were also excluded to allow focus on the Australian context; and also for consistency as characteristics of what defines a regional, rural or remote university campus can vary across countries. Reference lists were examined, and a supplementary Google Scholar search was undertaken, which together resulted in the identification of 5 additional relevant papers, with 12 papers^8^, ^9^, ^15^, ^16^ included in the narrative review. These 12 articles were then analysed, with the data synthesised and categorised into domains. Using an evidence table as outlined by Green et al^14^ the following data were extracted from the studies: author/s, year of publication, sample, study design, and relevant results, and are presented in Table 3.

**TABLE 3:** Evidence table

Reference	Sample	Design	Results	Comments
Birks et al^18^	18 satellite campus students, 155 main campus students, all undergraduate nursing	Quantitative. Comparison of satellite vs main campus students. Surveys. Mann‐Whitney *U* tests used for comparisons	Provision of nursing education at a satellite site offers an equivalent experience to students at a main campus	Small sample size of satellite students
Christensen et al^19^	38 undergraduate nursing students	Quantitative student evaluation. 15‐item Likert‐scale questionnaire and 6 open‐ended questions. One‐way statistical analysis	Easier access to nursing‐specific and general academic and pastoral support was beneficial to students who participated in the program	Small sample size
Croxon & Maginnis^33^	n/a (discussion paper)	Discussion paper. Student characteristics, characteristics of remote campus learning, university experiences	Students studying at rural campuses have different characteristics compared with regional and metropolitan areas. The student experience is also different, and students might need more support. The benefits of student retention and successful graduating nursing students can benefit their communities	
Felton‐Busch et al^21^	Action research—the 8 authors were the participants	Action research design underpinned by critical Indigenous methodology. Observation/creation of a guideline for culturally appropriate mentoring circles	Students studying at remote campuses within Indigenous communities need tailor‐made support programs to assist with academic and personal issues, and culture must be considered when planning these programs	
Gum^17^	8 undergraduate nursing students	Quantitative. Online survey closed‐ and open‐ended questions	Remote students face a range of barriers and enablers for successful rural study. Includes student perceived levels of support received, preparation for practice, career goals and future employment intentions	Small sample size
Maginnis and Croxon^16^	38 undergraduate nursing students and graduates	Quantitative. Questionnaire about family, study situation and campus experience, including satisfaction with teaching quality, modes of delivery and services provided to students	Both academic and non‐academic services are important to students' learning experiences at rural campuses. Students prefer face‐to‐face lectures, and traditional university services, for example library, social activities	Small sample size
Mills et al^22^	11 undergraduate nursing students, 68 artefacts	Qualitative. Action research design underpinned by critical Indigenous methodology. A 'storyline' was developed from the artefacts, including notes, activity outcomes, worksheets, posters, interview transcripts, meeting minutes	Increased confidence, communication, able to address barriers to study. Universities in similar contexts should create culturally appropriate support for Aboriginal and Torres Strait Islander students	
Nugent et al^8^	Data included from staff at 24 remote campuses in Australia	Quantitative. Survey, designed to capture no. of enrolled undergraduate nursing students, graduates and students expected to complete that year. Data analysed; frequencies calculated using SPSS	Lists several enablers for successful regional and remote nursing study	Exact number of responses unknown
Penman & White^15^	18 participants (10 mentors and 8 mentees, all undergraduate nursing students)	Open‐ended questionnaire. Student mentors and mentees evaluated a peer‐mentoring program at the university	Mentoring had a positive effect personal and professional growth of both mentees and mentors who participated	Small sample size, low response rate
Playford et al^9^	49 rural, 100 urban nursing graduates	Quantitative. Survey. Chi‐square tests using SPSS, grad numbers mapped to locations using GIS software	Comparison of rural vs. urban nursing students. Brief discussion of barriers and enablers to higher education and regional university study. Rural school nurses nearly twice as likely to work rurally upon graduation	
Usher et al^23^	n/a (discussion paper)	Discussion paper. Overview of the first couple of years of the JCU nursing program on Thursday Island	Importance of providing academic support to students in a remote Indigenous community context like the Torres Strait. Culturally specific barriers and enablers for Aboriginal and Torres Strait Islander students. Culturally appropriate academic support strategy	
Wirihana et al^20^	21 nursing academics working at satellite campuses	Qualitative. Phenomenological study. Colaizzi's framework. One‐hour interviews	Overall, lecturers at rural campuses reported that teaching at rural campuses was a different teaching experience, based on available resource, support they provided to students and relationship with main campus	

## 3 RESULTS

Seven studies^8^, ^9^, ^16^, ^17^, ^18^, ^19^ included in the review used quantitative methodology, consisting of surveys and open‐ended questionnaires; 3 studies^20^, ^21^, ^22^ used qualitative methods, including phenomenological and action research design; and there were 2 discussion papers.^16^, ^23^ Eleven of the papers focused on the student experience or outcomes, while one focused on the experiences of nursing academics teaching at satellite campuses across Australia. Three papers investigated student support strategies at the same satellite campus based in the Torres Strait Islands; their data were analysed within this cultural context. Studies using quantitative data collection methods were limited by small sample sizes, which might reflect the characteristics of satellite campuses. Three domains were prominent: student characteristics, barriers and enablers to studying nursing at a satellite university campus; teaching strategies and learning experiences; and academic and pastoral support. The literature review findings will be discussed under these domain headings.

### 3.1 Student characteristics, barriers and enablers

#### 3.1.1 Student characteristics

When compared to metropolitan university campuses, satellite campus nursing student cohorts are more likely to be first‐generation university students.^16^ Similar to their metropolitan counterparts, the majority of satellite campus nursing students are women, who are more likely to be mature‐aged. Satellite campus students are also more likely to come from rural areas^8^, ^15^ and from culturally and linguistically diverse backgrounds, including Aboriginal and Torres Strait Islander cultures.^15^, ^16^, ^21^, ^22^, ^23^

#### 3.1.2 Barriers

Five papers discussed barriers that satellite campus nursing students might face.^15^, ^16^, ^17^, ^20^, ^23^ Experiences of poor support from main university campus administration staff; negative perceptions of education delivery methods such as videoconference; and a lack of educational resources and services such as library and computing facilities to support study can have a negative influence on the student experience and lead to increased withdrawal from courses.^16^, ^17^ Internal factors that can affect satellite campus students’ ability to study effectively include a lower socio‐economic status (SES), rates of which increase with rurality.^16^, ^20^ Students might also have poorer information literacy and lower levels of preparedness for studying at university compared with those living in metropolitan areas.^16^, ^23^ As satellite campus students are often mature‐aged, they also need to balance the demands of study and clinical placements with work and family obligations.^15^, ^16^ Overall, satellite campus students are more likely than main campus students to be considered ‘at risk’ for poor academic results and attrition.^16^

Usher et al^23^ elaborated on the barriers faced by satellite campus nursing students to include culturally specific barriers often faced by Aboriginal and Torres Strait Islander students. These include financial, family and cultural expectations; limited space to study at home due to living with extended family members; unsympathetic university staff; culturally exclusive or insensitive university curricula, the ‘alien’ environment of university; differing learning styles of Indigenous students; and a lack of preparedness for all aspects of university learning.^23^

#### 3.1.3 Enablers

Four articles described enabling factors for satellite campus nursing students.^8^, ^9^, ^17^, ^19^ Having access to local tertiary education opportunities is an important enabler for remote and regional people to study nursing.^8^ Reduced entry requirements can encourage prospective students to apply for study at satellite campuses, and Australian Government initiatives including financial assistance might motivate rural and remote students to consider higher education and a nursing career, and help students avoid financial stress.^8^ Internal enabling factors such as not needing to relocate to access education is described as reducing the emotional, social and financial costs of moving away from home.^9^ This allows for continued support from friends and family, including supporting study, emotional support and child minding.^8^, ^17^, ^19^ Many regional and rural areas in Australia are more affordable to live in, which can also reduce the barrier of financial stress.^8^ Nursing students can also gain support and respect from their local community, which might further motivate them to continue their study.^17^

### 3.2 Teaching strategies and learning experiences

Six of the 12 articles discussed teaching strategies and learning experiences.^16^, ^17^, ^18^, ^20^, ^23^ Methods of nursing education delivery at satellite university campuses consistently aimed to provide an equivalent study experience. Students were enrolled as internal students in the same subjects and were required to complete assessment tasks identical to those studying at the main campus.^17^, ^18^, ^20^, ^23^ Local course coordinators facilitated classes and provide support; however, lectures and tutorials were often delivered by videoconference, or video‐ or audio recording, which were not considered ideal by students.^16^, ^17^, ^18^, ^23^ Campuses also arranged face‐to‐face teaching, either by regular visiting lecturers, transporting students to the main campus fortnightly, or intensive residential blocks at the main campus once per semester.^17^, ^18^, ^23^ Gum^17^ describes course delivery at the Flinders University Rural Clinical School, where students initially undertook fortnightly face‐to‐face classes with a double load to reduce travel requirements of lecturers from the main campus, located 4 hours' drive away. This was later replaced with videoconferencing to allow a more evenly spread content load for students, with an aim to deliver a study experience more equivalent to the main campus.

Factors that had a negative effect on student learning experiences included teaching methods that did not involve face‐to‐face teaching, especially audio and video recording of lectures. These non‐traditional education methods were described in the literature as ‘significantly negative factors’,^16^ ‘not a success’,^17^ and some students hesitated to ask questions during videoconferenced lectures due to shyness.^23^ Students also reported a lack of resources, study materials, library services, computing facilities and support from main campus staff.^16^, ^17^ However, students viewed smaller class sizes, a common trait of satellite university campuses, extremely positively, often commenting that this encouraged closer relationships and a supportive network with other students.^16^, ^17^ Students valued the face‐to‐face teaching they received and enjoyed familiarity and positive relationships with academic staff.^16^ In a study comparing satellite and main campus nursing students, Birks et al^18^ found no statistically significant difference in student satisfaction or assessment scores, which indicated that non‐traditional models of undergraduate nursing education at a satellite campus have the potential to offer an equivalent, enjoyable learning experience.

In a discussion piece, Croxon^16^ described staff at small university campuses as more enthusiastic and empathetic, which can improve student learning experiences. This was also found in a study conducted by Wirihana and Welch,^20^ who undertook a qualitative study of nurse academic experiences. Many challenges of working and teaching in rural locations were described, including feelings of being disconnected, forgotten and devalued by their metropolitan counterparts. Staff had a heavy reliance on information technology to support the delivery of education and were described as ‘jack of all trades’: they required generalist knowledge, filled multiple roles and adapted curriculum to the local context. Despite these challenges, staff had high job satisfaction and felt that they were ‘going the extra mile’ and ‘making a difference’, particularly with students from lower socio‐economic backgrounds, who had few support structures.^20^

Usher and colleagues described a nursing campus in the Torres Strait Islands that aimed to provide a supportive environment to improve Indigenous student retention and counter many of the issues common to satellite campus learning.^23^ In addition to the course content, students were provided with readings, additional contact time with academic staff, a structured timetable, and access to computer and Internet facilities. The presence of local staff increased familiarity between students and staff, helped to bridge language gaps and encouraged enrolment in the course.^23^ Importantly, the campus was developed with support from the local Indigenous community and other relevant stakeholders, an important consideration for university campuses in remote Australian communities.^23^

### 3.3 Academic and pastoral support programs

Five articles described academic and pastoral support programs, including mentoring programs^15^, ^21^, ^22^, ^23^ and an interprofessional nursing student support strategy.^19^ Penman and White^15^ evaluated a model of peer‐mentoring of nursing students at a satellite campus in South Australia. Initially being a structured model, it was replaced with a flexible, informal, ‘pop‐up’ model. Mentees and mentors could meet in various locations and communicate via phone and email, and mentors nominated times for consultations. It was designed to be student‐driven, with the mentee‐mentor partnership to be instigated and maintained by the mentee.^15^ Mentees could request assistance with nursing‐specific topics such as drug calculations and biosciences, academic skills such as writing and time management, and personal issues such as study/life balance.

Students reported that the peer‐mentoring service improved their motivation, helped to allay their fears regarding study and helped them to overcome learning difficulties. Benefits listed included improved academic performance, enhanced knowledge, greater confidence, and reduced isolation and self‐doubt.^15^ Mentors enjoyed sharing knowledge and skills, developing their teaching and mentoring skills and solidifying their own nursing knowledge. Both mentees and mentors benefitted from personal and professional development by participating in the peer‐mentoring program.^15^ Some negative feedback from mentees included no response to requests for support, difficulty with the mentor's personality, hesitation to ask for help and being unaware of the program. Mentors also reported difficulties balancing peer‐mentoring with their own study load, some were not approached at all, and a perceived poor response to mentor initiatives.^15^

Christensen et al^19^ evaluated an interprofessional approach to nursing student support at a satellite university campus, called ‘Nursing Tree Time’. The interprofessional team was located in the library and consisted of a nursing academic, who could provide nursing‐specific learning support; an academic skills advisor, to improve study skills and academic literacy; and a student counsellor, to provide financial advice and assist with time management, goal setting and pastoral support.^19^ Students consistently responded positively to the support received from the nursing academic and library support services. Interestingly, pastoral support was often sought from the nursing academic rather than the student counsellor, and the role of friends and family for personal support was much more pronounced than that of the counsellor.^19^ Unfortunately, researchers later found that several students did not use the support program, as they did not use the library or were unaware of the program.^19^

### 3.4 Aboriginal and Torres Strait Islander student support programs

Usher et al,^23^ Felton‐Busch et al^21^ and Mills et al^22^ all described culturally appropriate mentoring programs that have taken place at the satellite nursing campus in the Torres Strait. Usher et al^23^ reported a partnership between the University and Queensland Health staff on Thursday Island to deliver the ‘Tidda Balla’ mentoring program. This program involved Indigenous hospital nursing staff mentoring undergraduate nursing students, to provide a positive role model and help them to integrate into the future nursing workforce. This program also strengthened the relationship between Queensland Health, the University and the local community.^23^

Felton‐Busch et al^21^ and Mills et al^22^ described a mentoring circle program implemented to provide a safe, culturally appropriate and supportive environment, with the aim of improving retention and learning experiences of Indigenous undergraduate nursing students. Many of the students enrolled in the nursing course were not practically or emotionally prepared to cope with the ‘foreign environment’ of university.^22^ The mentoring circles allowed students to identify issues they faced, which had a detrimental effect on their studies, and create strategies to improve or prevent these issues. These included physical issues, such as access to resources and suitable study spaces; cognitive issues, such as time management and computer skills; communication issues, with other students and with staff, particularly during videoconference lectures; and personal issues, such as balancing study with work and family commitments, and communicating the importance of study to family members.^21^, ^22^ Students formed a group identity, and the design of a shirt for participants reinforced feelings of group pride and inspired other students to join. The mentoring circles improved student self‐awareness, confidence and leadership skills, and equipped students with skills and knowledge required for tertiary study.^22^

## 4 DISCUSSION

The purpose of this review was to investigate methods of education and student experiences of teaching, learning and support at satellite university nursing campuses. The factors that enabled or challenged satellite campus student success varied somewhat from the more ‘traditional’ students enrolled at metropolitan university campuses, as rates of lower SES, poorer education levels and literacy increased with rurality.^11^, ^24^ This review found that nursing students studying at satellite campuses are more likely to be mature‐aged, often balancing university, financial and family obligations, with a larger proportion of school‐leavers with lower rankings, first‐generation students, Aboriginal and Torres Strait Islanders and women returning to the workforce. Despite these barriers, there is evidence that rural students and students with lower SES might in fact achieve greater academic success and be more motivated than other students.^25^, ^26^ Aboriginal and Torres Strait Islander students might experience a range of factors in addition to those listed above that challenge successful university completion, including low English proficiency, isolation, racism, poor preparation for university and academic demands, and lack of cultural understanding and support from academic staff.^26^ Aboriginal and Torres Strait Islander students might also have cultural and family obligations that will take priority over study, including sharing finances despite economic hardship, caring for extended family members, family illness, funerals and the grieving process.^26^, ^27^

Like any health science student, nursing students are required to learn a considerable amount of theoretical and practical knowledge, and this can be further challenged when students are studying in regional and remote areas. As technology improves, effective education delivery methods have become available allowing students to study at a distance, either online or through satellite university campuses, and thus gain access to quality education. The Australian satellite university campuses included in this review generally used non‐traditional ‘hybrid’ or ‘mixed‐mode’ forms of education.^28^ Effective teaching via distance can be achieved, although this might require the instructor to change their teaching style to be more inclusive of all participants. Factors such as tone, inflection, pausing for questions and awareness of common issues such as time delay might improve the quality of the lectures as perceived by students.^28^ Collaboration across locations should also be encouraged, with the use of cross‐campus discussion boards and group projects, which includes the satellite campus students in the larger student cohort.^28^ While it is important to maintain standards of education across university sites, the remote learning environment is unique, which can benefit students, including greater flexibility, more enthusiastic and empathetic staff, smaller class sizes and other local opportunities.^11^ However, frequent use of distance education methods such as videoconferencing can contribute to higher rates of student dissatisfaction.^29^ Regional areas generally have poorer or limited access to Internet services, and interruptions caused by technical failures are not uncommon.^29^ These issues can be compounded for Aboriginal and Torres Strait Islander university students living in remote communities, who might also experience poorer access to information and communication technology (ICT) compared with non‐Indigenous students.^30^ This includes access to computer facilities, software, printing and Internet connections at home or public spaces; computer literacy; and affordability of ICT resources and equipment.^30^

This review has identified that the introduction of mentoring groups to support satellite campus nursing students has the potential to reduce the negative effects that might be experienced by students studying at a satellite campus. Mentoring and support strategies for student nurses might improve student learning and understanding, and examination preparation, and allow mentees and mentors to form strong, supportive bonds.^31^ Peer‐mentoring in particular has the potential to benefit both mentee and mentor with their studies, as mentors are able to solidify their knowledge, and develop teaching and leadership skills.^31^ Mentors can help students by sharing their own experiences, offering support and providing reassurance.^32^ Vandal et al^32^ found that face‐to‐face meetings, rather than virtual contact, was the most effective way for mentors and mentees to establish a meaningful relationship. A flexible, unstructured mentoring program allowed mentors to be resourceful, and create their own, unique mentoring style. Finally, student mentors gained skills in self‐reflection, collaboration and listening, all of which are central to nursing and were perceived to be beneficial to their own professional development.^32^

## 5 CONCLUSION

There is evidence that any rural experience, including having rural background, and rural clinical placement appear to influence graduates’ choice to live and work rurally; however, attending a satellite campus located in regional, rural or remote areas of Australia might have the greatest influence. Despite the sometimes problematic nature of learning at a satellite university campus, the benefits greatly outweigh the disadvantages. Recognition of the issues faced by satellite nursing campus staff and students and learning from successful educational and support strategies has the potential to improve rural and remote student nurse learning outcomes and increase retention. Supporting rural students who already have a vested interest in rural and remote communities and people can improve the sustainability of rural nursing workforce, leading to more positive health outcomes for rural and remote populations.

## CONFLICT OF INTEREST

Catherine Hays, Susan Devine, Nualnong Wongtongkam and Beverley Glass declare that they have no conflict of interest and have received no funding to conduct this research.

## AUTHOR CONTRIBUTIONS

Catherine Hays: Conceptualisation (lead); data curation (lead); investigation (lead); formal analysis (lead); methodology (lead); project administration (lead); writing—original draft (lead); writing—review and editing (equal). Susan Devine: Conceptualisation (supporting); project administration (supporting); supervision (supporting); writing—review and editing (equal). Nualnong Wongtongkam: Supervision (supporting); writing—review and editing (equal). Beverley Glass: Conceptualisation (supporting); project administration (supporting); supervision (lead); writing—review and editing (equal).
